# Nuclear Phosphoinositides—Versatile Regulators of Genome Functions

**DOI:** 10.3390/cells8070649

**Published:** 2019-06-28

**Authors:** Enrique Castano, Sukriye Yildirim, Veronika Fáberová, Alžběta Krausová, Lívia Uličná, Darina Paprčková, Martin Sztacho, Pavel Hozák

**Affiliations:** 1 Department of Biology of the Cell Nucleus, Institute of Molecular Genetics of the CAS, v.v.i., Vídeňská 1083, 142 20 Prague, Czech Republic; 2 Biochemistry and Molecular Plant Biology Department, CICY, Calle 43, No. 130, Colonia Chuburná de Hidalgo, Mérida C.P. 97200, Yucatán, Mexico; 3 Department of Epigenetics of the Cell Nucleus, Institute of Molecular Genetics of the CAS, v.v.i., division BIOCEV, 252 50 Vestec, Czech Republic; 4 Microscopy Centre of the Institute of Molecular Genetics of the CAS, v.v.i., 142 20 Prague, Czech Republic

**Keywords:** phosphoinositides, cell nucleus, genome, gene expression

## Abstract

The many functions of phosphoinositides in cytosolic signaling were extensively studied; however, their activities in the cell nucleus are much less clear. In this review, we summarize data about their nuclear localization and metabolism, and review the available literature on their involvements in chromatin remodeling, gene transcription, and RNA processing. We discuss the molecular mechanisms via which nuclear phosphoinositides, in particular phosphatidylinositol (4,5)-bisphosphate (PI(4,5)P2), modulate nuclear processes. We focus on PI(4,5)P2’s role in the modulation of RNA polymerase I activity, and functions of the nuclear lipid islets—recently described nucleoplasmic PI(4,5)P2-rich compartment involved in RNA polymerase II transcription. In conclusion, the high impact of the phosphoinositide–protein complexes on nuclear organization and genome functions is only now emerging and deserves further thorough studies.

## 1. Introduction

Phosphatidylinositol (PI) is a negatively charged glycerol-based phospholipid with a hydrophobic acyl tail and a hydrophilic inositol head. The hydrophobic acyl tail composition depends on the organism, as well as the tissue. In animals, the characteristic feature is a high content of stearic and arachidonic acids. All the stearic acid is linked to position sn-1, and all the arachidonic acid is linked to position sn-2. In plants, the main tail composition is of palmitic acid, which is the main saturated fatty acid in position sn-1, while linoleic and linolenic acids are the main unsaturated components in position sn-2. Similarly in yeast, palmitic acid is in position sn-1, with oleic and palmitoleic acids in position sn-2 [1,2].

The inositol ring, or cyclohexane-1,2,3,4,5,6-hexol, can be reversibly phosphorylated at the 3′, 4′, and 5′ positions, generating seven different phosphorylated phosphoinositides—phosphatidylinositol 3-phosphate (PI(3)P), phosphatidylinositol 4-phosphate (PI(4)P), phosphatidylinositol 5-phosphate (PI(5)P), phosphatidylinositol 3,4-bisphosphate (PI(3,4)P2), phosphatidylinositol 3,5-bisphosphate (PI(3,5)P2), phosphatidylinositol 4,5-bisphosphate (PI(4,5)P2), and phosphatidylinositol 3,4,5-trisphosphate (PI(3,4,5)P3). The importance of phosphoinositides in the regulation of important cytoplasmic processes, such as cell proliferation and cell death, regulation of microtubules and microfilaments, membrane dynamics, cell architecture, and motility or modulation of ion channels and transporters, is well established and was reviewed elsewhere [3,4,5,6,7,8,9,10,11,12]. Studies describing the nuclear localization of phosphoinositides and enzymes that synthesize them opened new horizons in the field of cell biology. However, we still lack the knowledge to answer some basic questions. Are nuclear phosphoinositides synthesized within the nucleus or transported to this compartment from the cytoplasm? How are these highly hydrophobic molecules retained within the nucleus, which is devoid of membranous structures? What is the mechanism of their action? In this review, we address these questions with a special emphasis on PI(4,5)P2, which was identified as an important regulator of a wide range of nuclear processes.

## 2. Transport of Phosphoinositides into the Nucleus

To answer the question about the origin of intranuclear phosphoinositides, one could consider that cytoplasmic phosphoinositides translocate to the nucleus just before the nuclear envelope reassembles during telophase, and then become enclosed by the newly formed nuclear envelope. For example, it was shown that nucleolar PI(4,5)P2 remains bound to nucleolar organizing regions (NORs) during mitosis [13]. On the other hand, the PI(4,5)P2 pool from nuclear speckles translocates to the cytoplasm and accumulates in mitotic interchromatin granules (MIGs). MIGs remain cytoplasmic even after the formation of the nuclear envelope in daughter cells [14] and their components enter the nuclei sequentially in late telophase [15]. So far, there is no evidence of specialized nuclear transporters for phosphorylated forms of PI. However, two isoforms of phosphatidylinositol transfer proteins (PITPs) which mediate cytoplasmic transport of PI were found in the nucleus [16,17,18].

**Figure 1:**
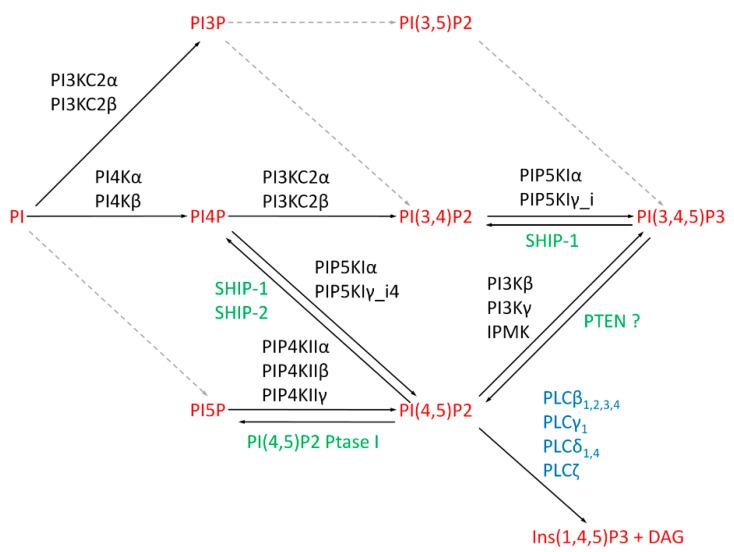
Known pathways of nuclear phosphatidylinositol and phosphoinositides. Depicted known phosphatidylinositol kinases in black and phosphatases in green, as well as their substrates (red). Black arrows indicate phosphorylation or dephosphorylation catalyzed by the known nuclear enzymes. Gray arrows show other potential pathways which might occur in the nucleus, but responsible nuclear enzymes are not yet confirmed. The scheme summarizes published data described in the text.

We may speculate that PI is imported to the nucleus, where it is subsequently phosphorylated. Indeed, many enzymes involved in phosphoinositide metabolism—kinases, phosphatases, and phospholipases—are present in the nucleus (Figure 1).

## 3. Metabolic Pathways for Nuclear Phosphoinositides

Due to the lack of tools for in vivo visualization of phosphoinositide synthesis, researchers demonstrated the intranuclear metabolism of phosphoinositides using in vitro studies. Smith and Wells [19] observed the enzymatic activity and incorporation of [γ32P]-ATP into PI(4,5)P2 in isolated nuclear membranes from rat liver cells. Cocco et al. [20] showed that phosphoinositides can also be formed within the nucleus regardless of membranous structures. They observed the incorporation of radiolabeled phosphate into phosphatidylinositol mono- and bisphosphate, the latter one presumably being PI(4,5)P2 [20]. Further observations made with the addition of exogenous substrates, PI, PI(4)P, and 1,2-diacylglycerol (DAG), to membrane-depleted nuclei and radiolabeled ATP showed reconstitution of the majority of lipid phosphorylations [21]. Payrastre and collaborators showed that nuclear matrices isolated from NIH 3T3 fibroblasts or rat liver cells contain PI 4-kinase, PI(4)P 5-kinase, DAG kinase, and PLC activities [22]. In addition, there is a dynamic metabolism of phosphoinositides in the nucleus, which is regulated during progression of the cell cycle [23,24,25,26]. It was shown that the amount of nuclear PI(4,5)P2 in synchronized HeLa cells decreases by 66% during the synthesis (S) phase [23]. In opposition to this, another study using synchronized murine erythroleukemia cells showed that the overall nuclear PI(4,5)P2 level does not change upon entry into the S phase, while the incorporation of radioactive phosphate into PI(4,5)P2 increased six-fold indicating a rapid metabolic turnover of PI(4,5)P2 within the nucleus [24]. This is consistent with the observation that retinoblastoma protein (pRb), which regulates the progression from the gap 1 (G1) to S phase, stimulates PIP5KIα activity and, therefore, also formation of PI(4,5)P2 in vitro [27,28], thereby regulating PI(4,5)P2 levels during the cell cycle. 

Enzymes able to subsequently generate almost all phosphoinositides species were detected in the nucleus. Nuclear PI3KC2α and PI3KC2β kinases can phosphorylate PI and PI(4)P at the 3′ position of the inositol ring to form PI(3)P and PI(3,4)P2 [29,30]. PI3KC2α is enriched in nuclear speckles and becomes phosphorylated upon RNA polymerase II (RNA pol II) inhibition by α-amanitin [29]. Although the role of PI3KC2α phosphorylation is unknown, this observation suggests a role of this kinase, or its products PI(3)P and PI(3,4)P2 in pre-messenger RNA (mRNA) processing. Nuclear PI3KC2β preferentially uses PI over PI(4)P as a substrate [30]. Moreover, its activity increases at the gap 2 (G2)/mitosis (M) transition in human promyelocytic leukemia HL-60 cells, indicating a role of PI(3)P in cell-cycle progression [25].

PI(4)P can be synthesized by two kinases, PI4Kα and PI4Kβ, which both phosphorylate PI at the 4’ position. Their activities were confirmed in the nuclei of human, mouse, rat, and yeast cells [31,32,33]. Moreover, Src-homology 2 containing inositol 5-phosphatases (SHIP-1, SHIP-2) hydrolyze the phosphate group of PI(3,4,5)P3 at the 5’ position to form PI(3,4)P2 [34,35]. SHIP2, phosphorylated at S132, also dephosphorylates PI(4,5)P2 and generates PI(4)P [36]. Phosphorylated forms of PI4Kβ and SHIP-2 localize to nuclear speckles. Moreover, PI4Kβ accumulates in nuclear speckles after RNA pol II inhibition by α-amanitin [36,37,38,39]. The detection of another PI(4)P synthesizing kinase, PI4Kα, implies localization of PI(4)P also in nucleoli [33].

A nuclear kinase that phosphorylates the inositol ring at the 5’ position is yet to be discovered, but nuclear PI(5)P can be formed by the type I phosphatidylinositol (4,5)-bisphosphate 4-phosphatase (PI(4,5)P2 4-Ptase I). Inversely, nuclear PI(5)P can be phosphorylated by the type II phosphatidylinositol 5-phosphate 4-kinase β (PIP4KIIβ) to PI(4,5)P2 [40]. Jones et al. [41] showed that PIP4KIIβ is inhibited upon cellular stress, resulting in the accumulation of PI(5)P in the nucleus. Similarly, PI(4,5)P2 4-Ptase I, which dephosphorylates PI(4,5)P2 to PI(5)P, translocates into the nucleus upon DNA damage [42], leading to accumulation of PI(5)P in the cell nucleus.

Both PI(4)P and PI(5)P may serve as precursors for PI(4,5)P2 synthesis. Type I phosphatidylinositol 4-phosphate 5-kinases (PIP5KI) utilize PI(4)P, whereas type II phosphatidylinositol 5-phosphate 4-kinases (PIP4KII) utilize PI(5)P as a substrate. Isoforms of both types, type I PIP5KIα and PIP5KIγ_i4, as well as type II PIP4KIIα, PIP4KIIβ, and PIP4KIIγ, localize to the nucleus [37,38,40,43,44,45]. Many of these enzymes colocalize with nuclear speckle markers [37,38,43]. Moreover, PIP5K1β can localize in both plasma membrane and vesicles in the perinuclei; therefore, it is likely to be active in the nucleus [46].

Nuclear PI(3,4,5)P3 is generated by phosphorylation of PI(4,5)P2 by class I PI3K kinases PI3Kβ and PI3Kγ or by inositol polyphosphate multikinase (IPMK) [47,48,49,50,51,52]. Moreover, PTEN phosphatase, which dephosphorylates PI(3,4,5)P3 at the 3’ position generating PI(4,5)P2 [53], localizes to both the cytoplasm and nucleus [54,55,56]. However, PTEN does not seem to act on PI(3,4,5)P3 in the nucleus [57].

In addition to kinases and phosphatases, PI(4,5)P2 is a substrate for phosphatidylinositol-specific phospholipase C (PI-PLC). PI-PLC cleaves PI(4,5)P2 and generates second messengers—inositol 1,4,5-trisphosphate (Ins(1,4,5)P3) and 1,2-diacylglycerol (DAG) [58]. The eukaryotic PI-PLC enzyme family consists of 13 isoforms, and eight of them were detected in the nucleus. The most studied PI-PLCβ1 contains a nuclear localization signal (NLS) in its C-terminus [59] and localizes to nuclear speckles [60,61]. Other subtypes of PI-PLCβ (PI-PLCβ2, β3, and β4) and other isozymes, such as PI-PLCγ1, PI-PLCδ1, PI-PLCδ4, and PI-PLCζ, were also confirmed to localize to the nucleus [59,62,63,64,65,66,67,68,69,70,71].

## 4. Localization of Nuclear Phosphoinositides

One of the biggest challenges in the field is the reduced number of tools for in vivo visualization of phosphoinositides. Therefore, antibodies and phosphoinositide-binding domains are widely used to detect phosphoinositides in cell nuclei via light and electron microscopy [72,73]. Gillooly et al. [74] used the FYVE domain of the receptor tyrosine kinase Hrs, a specific marker of PI(3)P, to identify PI(3)P in the dense fibrillar component of the nucleoli. We detected PI(4)P in the cell nuclei using the specific anti-PI(4)P antibody and PI(4)P-binding OSH1-PH domain. PI(4)P localizes to nuclear speckles and forms small foci in the nucleoplasm [72]. A specific antibody showed the presence of PI(3,4)P2 at the nuclear membrane [75]. Anti-PI(4,5)P2 antibodies (clone 2C11, clone KT10, and clone AM212) or the PI(4,5)P2-specific PH domain showed PI(4,5)P2 localization in nuclear speckles, nucleoli, and at the interface between the heterochromatin and the interchromatin space in the nucleus [13,14,37,72,76,77,78,79,80,81].

**Figure 2:**
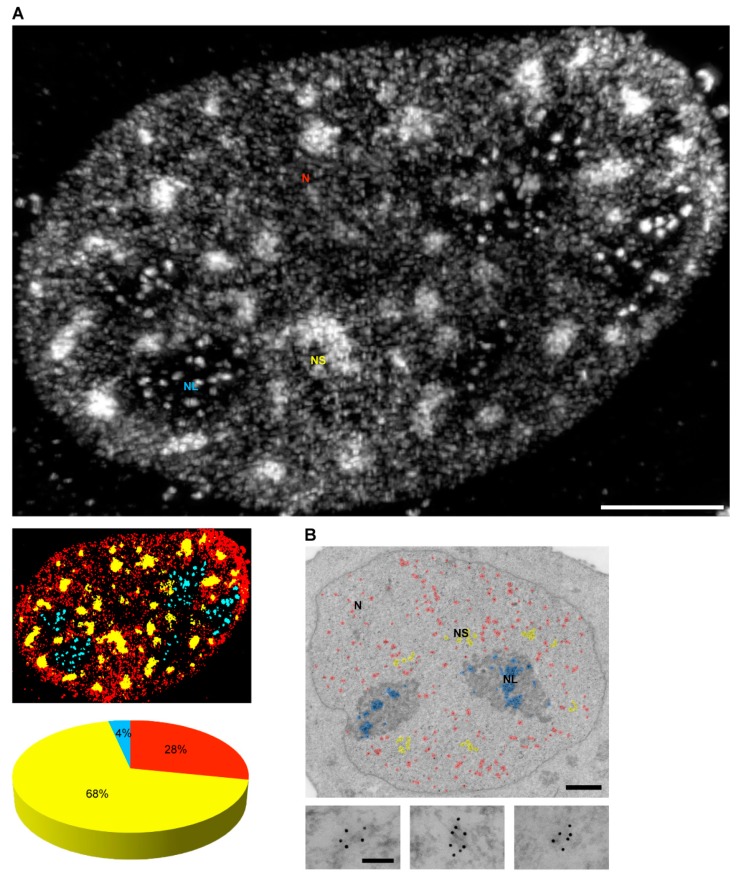
Nuclear lipid islets (NLIs)—a recently described nuclear compartment involved in RNA polymerase II (pol II)-dependent transcription. (**A**) Using super-resolution three-dimensional structured illumination microscopy (3D SIM), we revealed NLIs in the nucleoplasm as small foci labeled by anti-phosphatidylinositol 4,5-bisphosphate (PI(4,5)P2) antibody. Nuclear PI(4,5)P2 labeling reconstructed in 3D is presented here as a two-dimensional (2D) maximum intensity projection image. Using the NIS-Elements and MATLAB software, we determined the relative distribution of PI(4,5)P2 in nuclear speckles, nucleoli, and NLIs. The color-coded image and diagram presented below show PI(4,5)P2 in nuclear speckles in yellow, PI(4,5)P2 in NLIs in red, and the nucleolar PI(4,5)P2 in blue. (**B**) Transmission electron microscopy (TEM) demonstrates the localization of nuclear PI(4,5)P2 labeling in full detail. Color-coding is the same as in (**A**). NLIs of 40–100 nm are presented below in magnified view. Abbreviations in (**A**) and (**B**): N—nucleus, NL—nucleoli, NLIs—nuclear lipid islets, NS—nuclear speckles. The bars in (**A**) and (**B**, general view) are 1 µm; the bar in (**B**, magnified views) is 100 nm. Figure 2 is presented here with permission from Sobol et al. [75].

However, how are amphipathic phosphoinositides retained in the hydrophilic intranuclear environment? We recently showed that PI(4,5)P2 localizes to a specific, only recently described nuclear compartment—nuclear lipid islets (NLIs) (Figure 2). These globular structures have a diameter of 40–100 nm, with a surface formed mainly by PI(4,5)P2 molecules with hydrophilic heads presumably directed outward, while hydrophobic tails are directed inward. The core of NLIs is formed by carbon-rich compounds, likely lipids, whereas their periphery is associated with nucleic acids and proteins including RNA pol II large subunit, transcription factors, and nuclear myosin 1 (NM1), which contains a C-terminal PI(4,5)P2-specific pleckstrin homology (PH) domain [80]. Furthermore, PI(4,5)P2 can serve as a scaffolding platform which facilitates the formation of transcription factories, thus participating in the formation of nuclear architecture competent for transcription. The phosphoinositides can be plausibly required for phase separation in order to facilitate the formation of various nuclear compartments and molecular condensates separated from the surrounding environment, while having a reduced energy requirement to maintain this level of organization [82].

Another possibility is that the hydrophobic acyl tails interact with hydrophobic protein modifications of intranuclear proteins and hydrophobic protein pockets, or form bigger hydrophobic complexes. Such interactions can shield fatty-acid chains of phosphoinositides from the hydrophilic environment. Indeed, PI(4,5)P2 interacts with the myristoyl moiety of BASP1 transcription factor and anchors it at promoters of target genes [83]. While acyl chains bind to hydrophobic protein domains or larger phospholipid complexes, exposed phosphorylated inositol head groups of phosphoinositides can be metabolized by nuclear kinases and phosphatases. The head groups also bind the positively charged protein binding pockets. Phosphoinositides can, thus, form an interphase for protein–protein interactions [84]. Lewis et al. [85], using neomycin extraction, revealed that proteins with at least one positively charged lysine/arginine (K/R) amino acid motif interact with PI(4,5)P2, and identified over 300 nuclear proteins involved in processes such as DNA topological modification, chromatin remodeling, and RNA processing. The analysis of data from neomycin pull-down provided evidence of several phosphoinositide-binding motifs. One of them is a semi-conserved cluster of basic residues that correspond to the polybasic region (PBR) of certain proteins and can allosterically regulate histone binding specificity [86]. Another well-described phosphoinositide-binding motif is the plant homeodomain (PHD) finger domain. Binding of phosphoinositides to the PHD finger was shown to modulate chromatin association, and have a role in DNA damage response, ubiquitin signaling, and epigenetic regulation of gene expression [79,86,87,88].

## 5. Nuclear Processes Regulated by Phosphoinositides

To understand the different processes that involve nuclear phosphoinositides, a high-throughput study identified was carried out, and over 120 nuclear proteins showed binding to various phosphoinositides with different affinities [89]. These findings point rather toward multiple specific roles of particular phosphoinositides in nuclear processes. Below, we summarize proteins which are known to bind phosphoinositides in the nucleus and the roles of these interactions.

### 5.1. Anti-Apoptotic Signaling

Nuclear PI(3,4,5)P3 is an important molecule implied in cell differentiation and anti-apoptotic signaling [90,91,92]. PI(3,4,5)P3 binds directly to nucleophosmin (B23) in the nucleus, and their interaction is induced by nerve growth factor (NGF) treatment [92]. Moreover, protein kinase B (AKT), a PI(3,4,5)P3 effector protein, translocates to the nucleus after NGF stimulation in a PI3K-dependent manner [93,94]. In the nucleus, AKT interacts directly with B23 and protects it from degradation [95]. The PI(3,4,5)P3–B23 complex then mediates anti-apoptotic signaling and inhibits caspase-activated DNase, thereby protecting DNA from fragmentation [92]. The action of both phosphatases—SHIP-2 and phosphatase and tensin homolog (PTEN)—as well as the overexpression of PI(3,4,5)P3-binding mutant of B23, can inhibit NGF-induced anti-apoptotic actions [91,92]. Because it was reported that PTEN does not dephosphorylate PI(3,4,5)P3 in the nucleus [57], it is unclear whether PTEN can antagonize the NGF pathway through the decrease of PI(3,4,5)P3 level or via a different mechanism.

The direct binding of PI(3,4,5)P3 to the long non-coding RNA LINK-A, which facilitates AKT recruitment to PI(3,4,5)P3 resulting in the changes of AKT conformation and its activation, was described [96]. AKT also interacts with another PI(3,4,5)P3-binding protein, ErbB3-binding protein 1 (EBP1). EBP1 binds PI(3,4,5)P3 through the C-terminal polybasic motif. Although the role of PI(3,4,5)P3 in the function or localization of EBP1 is unclear, a mutation in the polybasic motif which reduces binding to PI(3,4,5)P3 is associated with cancer [97]. 

In addition, PI(5)P affects ubiquitin signaling in the nucleus as it stimulates the activity of the speckle-type POZ domain protein (SPOP)–Cul3 ubiquitin ligase complex. PIP4KIIβ kinase is a key regulator of (SPOP)–Cul3 ubiquitin ligase, and the mechanism behind stimulation of (SPOP)–Cul3 activity is most probably mediated by PI(5)P through a p38-dependent signaling pathway [98].

### 5.2. Chromatin Remodeling

Due to the negatively charged inositol head, PI(4,5)P2 interacts directly with positively charged histones H1 and H3 [99]. In vitro, RNA pol II transcription is inhibited by H1 [100,101,102]. This inhibition can be partially restored by the addition of PI(4,5)P2. These results suggest that the PI(4,5)P2 binding to H1 loosens the interaction of H1 with DNA and facilitates the progress of RNA pol II transcription in vivo. This process might be negatively regulated by phosphorylation of H1 by PKC [99].

Another mechanism of PI(4,5)P2 action in chromatin remodeling is through its direct interaction with Brg1, the ATPase subunit of the SWI/SNF-like BAF chromatin remodeling complex [103]. PI(4,5)P2 binds the BAF complex, targets it to chromatin, and facilitates changes in chromatin structure during T-lymphocyte activation [104].

PI(5)P can influence chromatin remodeling through proteins which do not contain PHD fingers [105]. Two components of the Sin3A corepressor complex, SAP30 and SAP30L, bind to phosphoinositides, preferably to PI(5)P, through a zinc motif which overlaps with the SAP30 DNA-binding site. Viiri et al. [105] proposed that PI(5)P binding displaces the Sin3A corepressor complex from DNA, leading to translocation from the nucleus to the cytoplasm.

### 5.3. Epigenetics

Histone acetylation reduces the positive charge of histones and loosens their association with negatively charged DNA. As a result, DNA is more accessible for transcription factors which can facilitate DNA transcription [106,107]. It was shown that chromatin acetylation is repressed by a process which requires PI(4,5)P2 and myristoylation of the transcriptional corepressor BASP1. It was shown that PI(4,5)P2 interacts with BASP1 through its myristoyl moiety and facilitates BASP1 interaction with histone deacetylase 1 (HDAC1). The BASP1–PI(4,5)P2–HDAC1 complex is then recruited to the promoter of target genes, where HDAC1 deacetylates histones and reduces promoter accessibility for RNA pol II transcription machinery [83]. 

It was shown that PI(5)P binds to the PHD finger domain of certain nuclear proteins and modulates their binding to chromatin, as well as having a role in DNA damage response, ubiquitin signaling, and epigenetic regulation of gene expression. In particular, PI(5)P is an important player in DNA damage response through its interaction with the inhibitor of growth protein 2 (ING2) [87]. Following DNA damage, ING2 expression is induced, which then stimulates acetylation of cellular tumor antigen p53, leading to enhancement of p53-dependent transcription, G1-phase cycle arrest, and apoptosis [108]. Under these conditions, the intranuclear level of PI(5)P is enzymatically elevated, which results in increased ING2 association with chromatin [41] and induction of apoptosis through the ING2–p53 pathway [42]. Very recently, the involvement of PIP5KIα and PI(4,5)P2 in the regulation of p53 was demonstrated. Choi et al. [109] showed that, upon DNA damage, PIP5KIα interacts with p53 and produces PI(4,5)P2, which in turn associates with p53 through its C-terminal domain (CTD). The binding of PI(4,5)P2 to p53 contributes to the recruitment of the small heat-shock proteins HSP27 and αB-crystallin, which bind to p53, thus promoting its stabilization.

Furthermore, in *Arabidopsis* plants, PI(5)P affects the level of H3K4me3 [110]. The *Arabidopsis* homolog of trithorax (ATX1) is a trimethyltransferase that acts on H3K4 at the promotor regions, binds to PI(5)P, and causes ATX1 detachment from promoters and translocation from the nucleus to the cytoplasm. These data suggest that the effect of PI(5)P binding to PHD domains can have different impacts on its binding partners [88].

Gelato et al. [86] described the regulation of ubiquitin-like PHD and RING finger domain-containing protein 1 (UHRF1) by PI(5)P. UHRF1 is a multidomain protein which binds unmodified histone H3 and recruits histone methyltransferases to methylate H3K9, thus establishing transcription repressive marks. Moreover, UHRF1 is able to bind H3K9me3 and, being in a complex with histone methyltransferases, maintains heterochromatin state [86]. PI(5)P binds directly to the polybasic region (PBR) in the C-terminal part of UHRF1, changes the conformation of UHRF1, and allosterically regulates its histone binding specificity. When not bound to PI(5)P, UHRF1 recognizes the unmodified histone H3 tail, while the PI(5)P–UHRF1 complex binds histone H3K9me3 [86]. Therefore, PI(5)P levels could regulate UHFR1 association with chromatin, thereby having an impact on the heterochromatic state of the genome.

### 5.4. Gene Expression

PI(4,5)P2 and PI(5)P regulate transcription during myogenic differentiation. A knock-down of PIP4KIIβ, resulting in accumulation of PI(5)P, increases myotube formation through modulating TATA box binding protein 3 (TAF3). Both PI(5)P and PI(4,5)P2 modulate TAF3 interaction with H3K4me3 and regulate expression of specific genes [111]. It was shown that the activity of class I PI3K is increased upon induction of granulocytic differentiation and affects growth factor stimulation of various cell lines or isolated nuclei [90,91,112].

Both PI(4,5)P2 and PI(3,4,5)P3 can interact with steroidogenic factor 1 (SF-1) [84,113], which regulates the transcription of genes involved in lipid and steroid metabolism, as well as in the regulation of cytoskeleton dynamics, cell cycle, and apoptosis [114]. PI(4,5)P2 or PI(3,4,5)P3 bind to the SF-1 sterol-binding pocket through their acyl chains and stabilize the tertiary structure of SF-1. SF-1 in a complex with PI(3,4,5)P3 displays significantly higher affinity for a coactivator peptide than in a complex with PI(4,5)P2 [84,113]. Therefore, the action of IMPK kinase or PTEN phosphatase can affect SF-1 activity and, thus, SF-1 target gene expression [84]. Additional work is required to define PTEN involvement and clarify its active role in the nucleus in this process as compared to earlier findings [57]. Furthermore, in *Caenorhabditis elegans*, depletion of PI(4,5)P2 results in chromosome structural changes which lead to various defects during meiotic progression. The altered chromosome structure is accompanied by the increased transcription activity, which might indicate the DNA-damage-driven apoptosis in the *C. elegans* gonad [115]. 

### 5.5. RNA Pol II-Dependent Transcription Initiation

**Figure 3:**
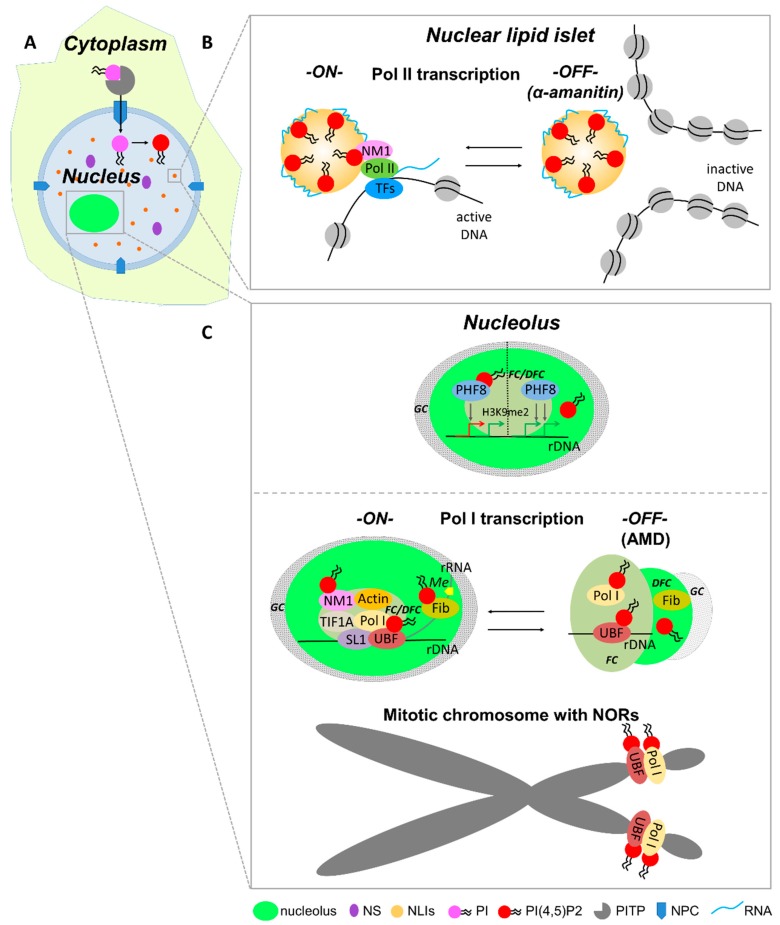
A schematic view of PI(4,5)P2 involvement in RNA pol I and II transcription on the NLI surface and the regulation of nucleolar processes by PI(4,5)P2. Based on published data [11,67,73,74,75]. (**A**) Phosphatidylinositol (PI) is transported into nucleus together with phosphatidylinositol transfer protein (PITP) and is converted to PI(4,5)P2 via phosphorylation by PI kinases (PIKs) and PI phosphate kinases (PIPKs). (**B**) PI(4,5)P2 is the major component of NLIs, where it may serve as an anchor for the assembly of transcription complexes. PI(4,5)P2 plays a role in promoting RNA pol II transcription together with transcription factor NM1. Upon inhibition of RNA pol II transcription via α-amanitin, the pattern and quantity of NLIs do not change, implying a role for NLIs as a structural platform for the transcription factories. (**C**) In the nucleolus, PI(4,5)P2 directly interacts with PHF8 and represses its function as H3K9me2 demethylase. The PI(4,5)P2-binding mutant of PHF8 has increased activity on ribosomal DNA (rDNA) promoter, which leads to the increased expression of rDNA. Also, PI(4,5)P2 is present at the promoter of RNA pol I, and depletion of PI(4,5)P2 leads to a significant decrease in transcription level. PI(4,5)P2 binds to upstream binding factor of RNA pol I (UBF) and fibrillarin in the fibrillar center (FC) and dense fibrillar component (DFC), respectively. Inhibition of RNA pol I transcription via drugs results in disassociation of PI(4,5)P2 from fibrillarin in the DFC; however, PI(4,5)P2 colocalizes with UBF and RNA pol I in the FC regardless of active transcription. Moreover, during mitosis, when the transcription is switched off, PI(4,5)P2 preserves its association with UBF and RNA pol I in nucleolar organizing regions (NORs). This suggests a structural role for PI(4,5)P2 in the formation and/or maintenance of core helix of NORs. FC—fibrillar centers, Fib—fibrillarin, DFC—dense fibrillar component, GC—granular component, Me—methyl group, NLIs—nuclear lipid islets, NM1—nuclear myosin 1, NPC—nuclear pore complex, NS—nuclear speckles, PI—phosphatidylinositol, PIP2—phosphatidylinositol 4,5-bisphosphate, PITP—phosphatidylinositol transfer proteins, Pol I—RNA polymerase I, Pol II—RNA polymerase II, SL1—selectivity factor 1, TFs—transcription factors of RNA pol II, TIF1A—transcription initiation factor of RNA pol I, UBF—upstream binding factor of RNA pol I.

**Figure 4:**
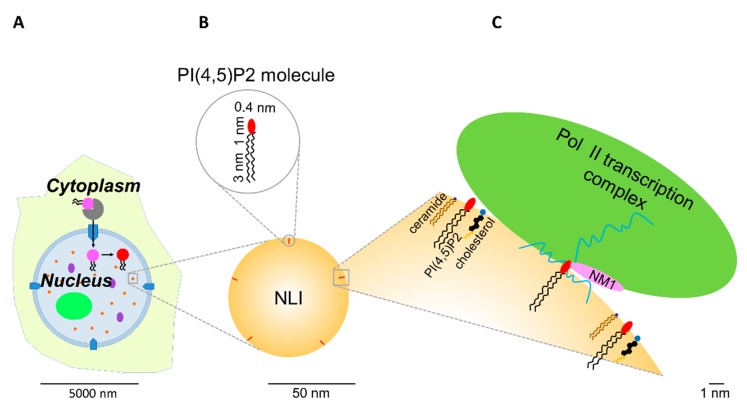
Detailed model of the surface of NLI. The model is depicted roughly in proportion to the real size of the molecules based on experimental data from Sobol et al. [75]. Abbreviations and colors used in this model are the same as in Figure 3. (**A**) is the same as in Figure 3. (**B**) Comparative size of NLI and PI(4,5)P2 molecule. (**C**) A part of the surface of NLI, composed of PI(4,5)P2, ceramide, cholesterol, and RNA, with the associated RNA pol II transcription complex, NM1, and nascent transcripts.

PI(4,5)P2 forms a complex with the active form of RNA pol II, as shown by immunoprecipitation with anti-PI(4,5)P2 antibody or a pull-down experiment with PI(4,5)P2-coupled beads [14,80,83]. However, there is still no evidence of a direct interaction between PI(4,5)P2 and RNA pol II. Our recent study suggests that PI(4,5)P2 can be bound to RNA pol II transcription machinery through a direct interaction with the transcription factor nuclear myosin 1 (NM1). In vivo transcription and pull-down assays showed that NM1 requires association with PI(4,5)P2 to form a complex with the active form of RNA pol II, thus maintaining transcription [80]. We showed that PI(4,5)P2 associates with actively transcribed genes, RNA pol II, the RNA pol II transcription factors, NM1, and nascent transcripts at the surface of NLIs (Figure 3 and Figure 4) [80]. Our data suggest that NM1 can tether the chromatin-remodeling complexes to NLIs supporting the transcriptionally competent chromatin state [80].

### 5.6. Pre-mRNA Processing

PI(4,5)P2 depletion from HeLa nuclear extract by anti-PI(4,5)P2 antibody inhibits pre-mRNA splicing in vitro. However, addition of PI(4,5)P2 itself into depleted extract does not restore splicing reaction. This indicates that not only PI(4,5)P2 but also its binding partners—small nuclear RNAs (snRNAs) and the active form of RNA pol II—are necessary to restore splicing activity [14]. However, the exact molecular mechanism behind this phenomenon remains unknown.

Once mRNA is matured, it is exported to the cytoplasm upon association with RNA-interacting protein complexes [116,117]. PI(4,5)P2 and PI(3,4,5)P3 directly interact with the N-terminal part of the export factor Aly/REF. A disruption of this interaction displaces Aly from nuclear speckles, and mRNA export is attenuated [118]. Furthermore, Wickramasinghe et al. [119] showed that the PI3K activity of IPMK and nuclear PI(3,4,5)P3 formation is essential for Aly binding to target mRNA transcripts, which code factors involved in the repair of DNA double-strand breaks by homologous recombination.

### 5.7. rDNA Modification and RNA Pol I Transcription

Transcription of rDNA by RNA pol I takes place in the nucleolus in specific compartments where tandem repeats of pre-ribosomal RNA genes are assembled [120,121]. The upstream binding factor (UBF) and the promoter selectivity factor (SL1) form a complex, which is recruited to the rDNA promoter and facilitates the initiation of RNA pol I transcription [122]. UBF binds not only to the rDNA promoter but also to the enhancer and the transcribed region of rDNA genes [123,124,125,126,127,128]. We showed that PI(4,5)P2 binds to UBF enhancing its binding to the rDNA promoter. Moreover, the depletion of PI(4,5)P2 from HeLa nuclear extract decreases the level of RNA pol I transcription in vitro. The decrease can be partially restored by the addition of PI(4,5)P2 into the transcription reaction. The depletion of PI(4,5)P2 after the transcription initiation did not cause any significant change in the transcription [78]. These data suggest that the PI(4,5)P2–UBF interaction might be required for the association of the transcription initiation complex with rDNA and activation of RNA pol I transcription (Figure 3). Since UBF can trigger the formation of pseudo-NORs by binding to specific regions of rDNA [124], we propose that PI(4,5)P2 can modulate binding of UBF to rDNA sequences and, therefore, participate in NOR formation. In accordance with this model, we showed that PI(4,5)P2 interacts with RNA pol I subunits and UBF regardless of active transcription, indicating a structural role for PI(4,5)P2 in the formation and maintenance of NORs [13] (Figure 3). In the in vitro experiments using the purified histones from *Brassica oleracea*, we showed that PI(4,5)P2 degradation by PLC results in increased histone binding to the rDNA promoter [129]. This suggests an additional mechanism of the RNA pol I transcription regulation by PI(4,5)P2.

Our group showed that PI(4,5)P2 also binds to another nucleolar protein—fibrillarin [78]. Fibrillarin functions in pre-rRNA processing, modification, and ribosomal assembly and, therefore, is crucial for cell vitality [130]. Moreover, the depletion of fibrillarin impairs the normal embryo development [131]. PI(4,5)P2 binds to fibrillarin in the dense fibrillar component of the nucleolus and modulates the binding of fibrillarin to pre-rRNA [78]. The interaction between fibrillarin, PI(4,5)P2, and rRNA may provide a link between the transcription initiation process and the processing machinery (Figure 3).

Moreover, we showed that PI(4,5)P2 contributes to the regulation of rRNA gene expression not only at the transcriptional, but also at the epigenetic level [79,132]. PI(4,5)P2 directly interacts with PHD finger protein 8 (PHF8) histone lysine demethylase through its C-terminal K/R-rich motif. PI(4,5)P2 binding promotes conformational changes in PHF8, represses its H3K9me2 demethylase activity and, thus, regulates expression of pre-rRNA genes [79] (Figure 3).

## 6. Conclusions

Taking together, all these data indicate that phosphoinositides play multiple specific roles in the nuclear processes depending on their binding partners and physiological state of a cell.

In order to regulate the nuclear functions, the processes in the cell nucleus are spatially and temporarily organized. It is, therefore, crucial to understand molecular interactions leading to the formation of the intranuclear order, such as spatial maintenance of chromosomal domains, transcription-dependent movements of gene loci in nuclear volume, and various epigenetic effects regulating gene expression. This review summarizes the origin and essential functions of nuclear phosphoinositides, and describes multiple roles of PI(4,5)P2 in the regulation of gene expression, which are currently emerging.

The available data suggest that PI is transported to the nucleus via PITPs [16,17,18], where it can be consecutively phosphorylated to form nuclear phosphoinositides. However, a redundant system may exist to transport and maintain the level of nuclear PIs, since changes of expression of PITPs do not dramatically affect the amount in the nucleolus [133]. The activity of phosphatidylinositol kinases/phosphatases and, thus, the nuclear phosphoinositide levels are dynamically regulated during the cell cycle and under various cell conditions [23,24,25,26,41,42,90,91,111,112,134,135].

Phosphoinositides interact with nuclear proteins, resulting in changes of protein conformation, localization, and activity. In such a way, phosphoinositides modulate a variety of important nuclear processes such as chromatin remodeling and modification, DNA transcription, pre-mRNA processing, RNA export, cell-cycle regulation, and DNA damage response [13,14,38,41,76,77,78,79,80,83,85,86,91,92,98,99,103,104,111,113,118,119,134,136,137]. We suggest that PI(4,5)P2 and other phosphoinositides can act as an interface to facilitate the molecular interactions and reorganizations of the nuclear/nucleolar complexes (Figure 3 and Figure 4). A possible advantage of this boundary is a rapid turnover of phosphoinositides by kinases and phosphatases within the nucleus, allowing fast regulation of transcription and other nuclear processes. Differential phosphorylation of phosphoinositides can result in a higher or lower affinity for their interacting molecules, thus modulating the activity and the composition of the particular complexes.

The compartmentalization of the nucleus and the existence of the different pools of the same phosphoinositides might allow local fine-tuning of the metabolism of the distinct phosphoinositides in the specific nuclear compartment at exact time points. This permits affecting only the particular process with no or minimal impact on the others. There is growing evidence that the organization of the membraneless intranuclear compartments like nucleoli, nuclear speckles, paraspeckles, and chromatin domains is due to phase separation through specific interactions between different parts of the complex molecules like phosphoinositides, proteins, and nucleic acids [138,139,140,141,142,143,144,145]. We already suggested that NLIs might represent a phase-separated system as well [80]. Recently, our suggestion was endorsed by Boehning et al. [142], who showed that the CTD of RNA pol II phase separates and is crucial for the formation of transcription factories. We believe that the impact of the nuclear phosphoinositides on the spatial organization and functions of nuclear and nucleolar subdomains is of a great importance and needs to be elucidated further. Available data suggest that the disruptions of the phosphoinositide–protein complexes and their impaired functions may affect the general nuclear organization. That is why we expect the exponential and qualitative progress in the field to bring about new key findings and discoveries. However, the functional redundancy of the enzymes involved in the nuclear phosphoinositide metabolism and the lack of in vivo phosphoinositide labeling are the biggest challenges currently being faced.
